# Mechanical features of endothelium regulate cell adhesive molecule-induced calcium response in neutrophils

**DOI:** 10.1063/1.5045115

**Published:** 2019-03-28

**Authors:** Yanhong Xu, Dandan Huang, Shouqin Lü, Yan Zhang, Mian Long

**Affiliations:** 1 Center of Biomechanics and Bioengineering, Institute of Mechanics, Chinese Academy of Sciences, Beijing 100190, China; 2 Key Laboratory of Microgravity (National Microgravity Laboratory), Institute of Mechanics, Chinese Academy of Sciences, Beijing 100190, China; 3 Beijing Key Laboratory of Engineered Construction and Mechanobiology, Institute of Mechanics, Chinese Academy of Sciences, Beijing 100190, China; 4 School of Engineering Science, University of Chinese Academy of Sciences, Beijing 100049, China

## Abstract

Atherosclerosis is caused by chronic inflammation associated with the adhesion of neutrophils and endothelial cells (ECs) that is mediated by their respective cellular adhesive molecules to stiffened blood vessel walls. However, the stiffness dependence of calcium flux on neutrophils remains unclear yet. Here, the effect of substrate stiffness by ECs on neutrophils' calcium spike was quantified when the individual neutrophils that adhered to the human umbilical vascular endothelial cell (HUVEC) monolayer were pre-placed onto a stiffness-varied polyacrylamide substrate (5 or 34.88 kPa) or glass surface. Our data indicated that E-/P-selectins and intercellular adhesion molecule 1 (ICAM-1) on HUVECs and β_2_-integrins, P-selectin glycoprotein ligand 1 (PSGL-1), and CD44s on neutrophils were all involved in mediating neutrophil calcium spike in a stiffness-dependent manner, in which the increase in substrate stiffness enhanced the calcium intensity and the oscillation frequency (spike number). Such stiffness-dependent calcium response is associated with the induced selectin related to β_2_-integrin activation through the Syk/Src signaling pathway, and F-actin/myosin II are also involved in this. Moreover, tension-activated calcium ion channels displayed critical roles in initiating stiffness-dependent calcium spike. These results provide an insight into understanding how the stiffening of vascular walls could regulate the calcium flux of adhered neutrophils, and thus the immune responses in atherosclerosis.

## INTRODUCTION

As chronic vascular inflammation, atherosclerosis presents increased arterial stiffening, which is a hallmark of atherosclerosis and also the consequence of many diseases such as diabetes and chronic renal compromise. In atherosclerosis, sub-endothelial matrices of arteries are much stiffer [∼28 kPa (Ref. 1)] than healthy matrices [ranging from 2.5 kPa to 8 kPa (Refs. 2 and 3)]. This artery stiffening results in an increased number of leukocytes penetrating the endothelium, accumulating at the inflammatory site and aggravating the vascular injury. Finally, these processes lead to vascular stenosis and sclerosis, and thus stiffen the vessel wall. In the past decade, monocytes and macrophages are known to be involved in the onset, progression, and manifestation of atherosclerotic lesions.^4^ Recently, neutrophils began to attract attention with respect to atherosclerotic lesion growth.^5,6^ Nevertheless, it still remains unclear how neutrophils might act on atherogenesis, progression, and atherosclerotic plaque destabilization.

Neutrophils are major constituents of the cellular immune system and serve as the first-line defense to invading pathogens. In atherosclerotic vessels, neutrophils are exposed to widely varied physiological and mechanical microenvironments including shear force, chemo-attractants, and mechanical properties (e.g., vessel stiffness and blood pressure), driving neutrophils to adapt and respond to the varied environmental challenges for functioning properly.^7^ Interestingly, neutrophils are able to sense the differences in both shear force and surface-bound adhesive proteins to trigger a well-defined cascade of neutrophil recruitment involving capturing, rolling, slow rolling, adhesion, intravascular crawling, and transmigration across the endothelial cell (EC) monolayer. At the molecular level, selectins and intercellular adhesion molecule 1 (ICAM-1) in endothelial cells as well as P-selectin glycoprotein ligand 1 (PSGL-1), β_2_-integrins (LFA-1, Mac-1) and CD44 in neutrophils are key adhesive receptors in these processes. For example, neutrophil rolling on E-selectin forms catch bonds with L-selectin that transduces high affinity β_2_-integrin bond formation with ICAM-1, inducing the neutrophil arrest on the inflamed endothelium under shear flow.^8^ E-/P-selectins contribute to the dynamics of neutrophil trans-endothelial migration by activating β_2_-integrins to high affinity *via* binding to their ligands in a differential way.^9^ While a body of evidence on immune cascade in atherosclerosis mainly focuses on the distinct molecular mechanisms and the roles of shear flow, the effect of vessel wall stiffening is rarely known.

Substrate stiffness is known to have a significant impact on cellular behaviors for both endothelial cells and neutrophils. In the past decades, attention has been focused on the effects of substrate stiffness on cell adhesion, migration and morphology. For example, ECs on a stiff substrate exerting larger traction forces have a more stretched and reorganized actin cytoskeleton than those on a soft substrate,^10–12^ and elasticity variation of human umbilical vascular endothelial cells (HUVECs) alters cell-cell adhesion through Myosin light chain kinase (MLCK)-dependent cell contraction.^13^ Neutrophil’s behaviors also depend on substrate stiffness, as exemplified by the fact that the neutrophil migration speed is biphasic with regard to substrate stiffness,^14^ and that neutrophils display increased spreading (from rounded to flattened morphology) with increasing substrate stiffness.^12,14,15^ Although how these mechanical cues individually affect ECs and immune cells is of significance, it is necessary to define the effects of the varied elasticity of the endothelium as a neutrophil “substrate.” Considering that neutrophils come in direct contact with the HUVEC layer but not the basement membrane or extracellular matrixc (ECM) under physiological conditions, there exists the complex interplay among the substrate stiffness, EC mechanics and polymorphonuclear neutrophil (PMN) responses in the cascade of neutrophil recruitment. In this regard, the endothelium could serve as a “substrate” for neutrophils, bridging the neutrophils that adhered to and the substrate underneath the endothelium.

Ca^2+^ is an intracellular second messenger and has long been known as the key events of cellular immunity, i.e., chemotaxis, phagocytosis and activation.^16,17^ Ca^2+^ concentration contributes to neutrophil activation and essentially regulates important cellular events in neutrophils including degranulation, chemotaxis, and migration. Changes in the spatial and frequency distribution of calcium spike affect multiple aspects of neutrophil activity.^16^ Recently, migrating neutrophils are found to exhibit the enriched calcium flux at the leading edge, and this intracellular Ca^2+^ response helps define the direction of cell movement.^16^ Meanwhile, cytoplasmic calcium concentration is a sensitive indicator of the cell activation level during the trafficking of neutrophils into sites of inflammation.^18^ However, it still needs to be deciphered how this calcium response varies with vessel wall stiffening in atherosclerosis.

Here, we focused on understanding how substrate stiffness affects neutrophil calcium response *via* pre-monolayered human umbilical vascular endothelial cells (HUVECs). We hypothesized that the matrix stiffening during atherosclerosis stiffens the ECs, which in turn alters the interactions between ECs and neutrophils *via* the cellular adhesive molecules involved. High stiffness allows further activation of β_2_-integrins through physical linkage of these molecules, and thus enhances neutrophil calcium response. To test this, neutrophil calcium flux was quantitatively compared among stiffness-varied polyacrylamide (PA) hydrogels or on glass, and the potential effects of selectins and ICAM-1s on ECs or PSGL-1, CD44 and β_2_-integrins on neutrophils were elucidated separately. The underlying signaling pathways of this stiffness-dependent regulation in calcium response were also discussed.

## RESULTS

### LPS-induced enhancement of cellular adhesive molecule expression

**FIG. 1.**
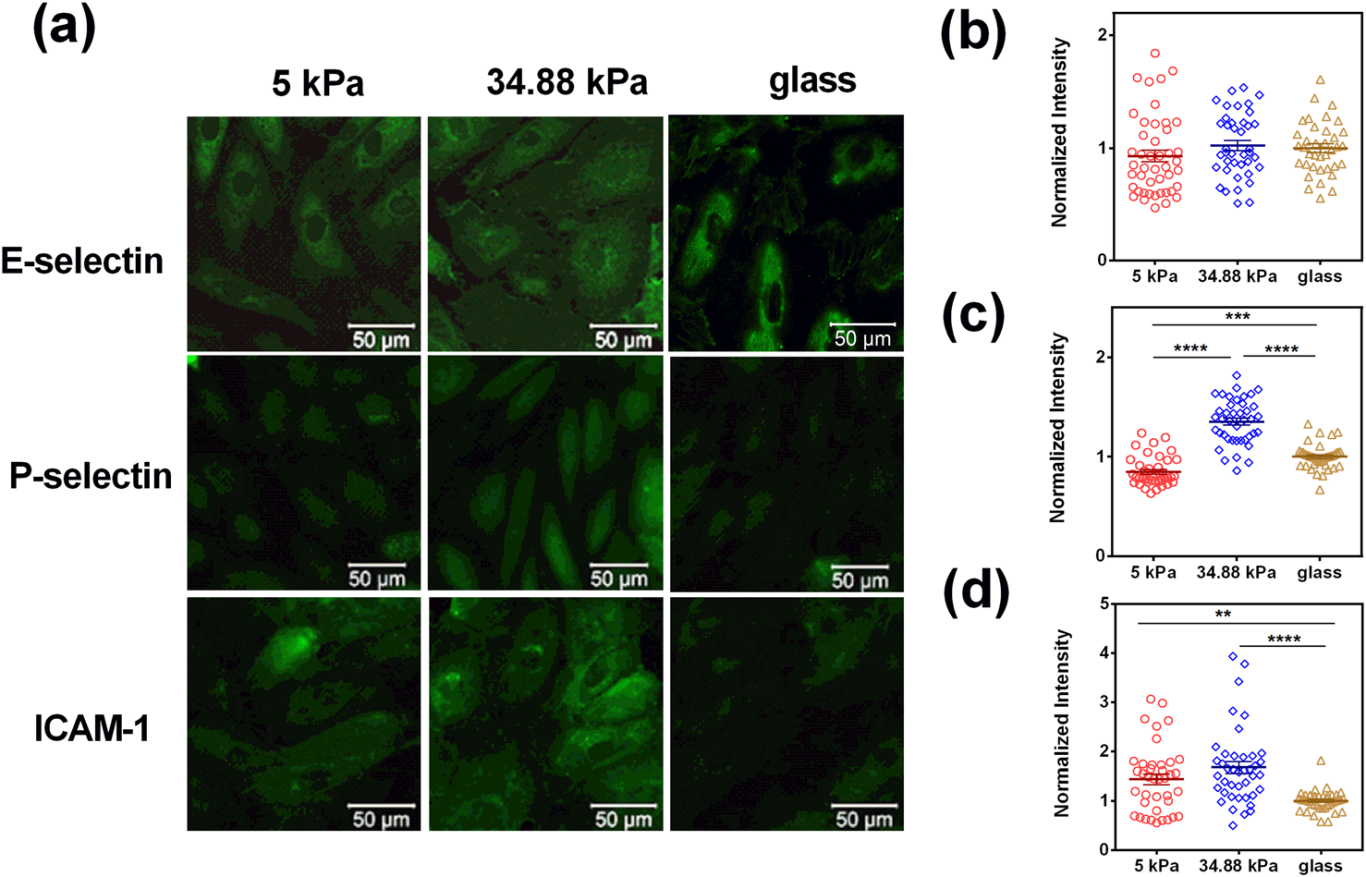
Expressions of E-/P-selectins and ICAM-1s on 4-h LPS-stimulated HUVECs at different stiffnesses. (a) Fluorescent images of the HUVEC monolayer at different stiffnesses treated with culture media containing 1 *μ*g/ml LPS for 4 h and then stained with respective mAbs. Normalized fluorescence intensities of E-selectins (b), P-selectins (c), and ICAM-1s (d) were obtained by dividing the average fluorescence intensity of each field of view (FOV) on PA gel by the value on glass in the same experiment. Data were collected from 3 independent experiments for a total of 37–45 FOVs for each condition. All data were compared using one-way analysis of variance (ANOVA) followed by Tukey's post-hoc test and presented as the mean ± standard error (SE). **p <* *0.01, ***p <* *0.001, and ****p <* *0.0001.

We first built up an *in vitro* cellular inflammation model by pre-culturing HUVECs on a PA gel with a thickness of 100 *μ*m to from a monolayer. To examine the potential involvement of adhesive molecules in the Ca^2+^ spike of neutrophils, the HUVECs were treated with lipopolysaccharide (LPS) for 4 h to induce the expression of selectins and ICAM-1s before tests, as previously reported.^9^ The 4-h LPS-treatment enhanced the expressions of E- and P-selectins and ICAM-1s on HUVECs compared to those quiescent cells without LPS treatment [supplementary material, Fig. S1(a)]. Fluorescence intensity analysis confirmed the above observations, yielding a 16.3-, 2.0- or 13.7-fold increase in E- and P-selectins, or ICAM-1 expression [supplementary material, Fig. S1(b)]. Moreover, this LPS-induced enhancement of cellular adhesive molecules was not positively correlated with substrate stiffness (Fig. 1). Specifically, E-selectin expression was indifferent at three stiffnesses, P-selectin expression was higher at 34.88 kPa, and high ICAM-1 expression was observed at both 5 and 34.88 kPa.

### HUVEC elasticity increase with increased substrate stiffness

**FIG. 2.**
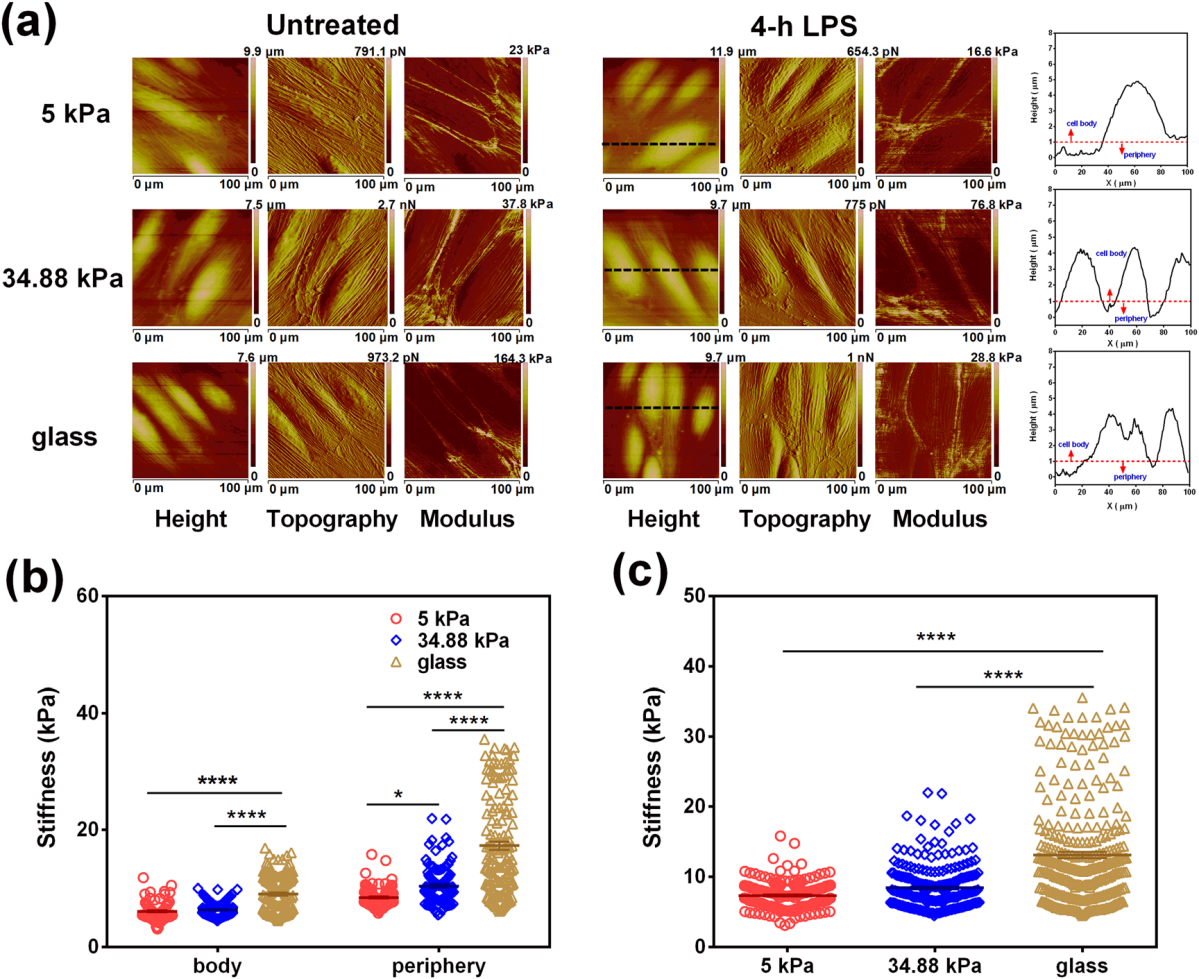
Global or local elastic moduli of HUVECs at different stiffnesses. (a) Height (1st and 4th columns), topography (2nd and 5th columns), and modulus (3rd and 6th columns) images of the intact or 4-h LPS-treated HUVEC monolayer obtained on 5- or 34.88-kPa PA gel, or a glass substrate using PFQNM-LC probes in QNM mode. Individual HUVECs were segregated into two regions by plotting an arbitrary line in the height image (*black line* in a typical 4th column) and defining from the quantified height curve the cell body as the region with height ≥1 *μ*m and the cell periphery as the one with height <1 *μ*m (*red line* in the right-most column). (b) The regional elastic moduli of the cell body and the cell periphery were quantified separately on different substrates. (c) Global elastic moduli of individual HUVECs were obtained by pooling all the data from the same condition and averaging them out on each substrate. Each AFM image in (a) is 100 × 100 *μ*m^2^. All the data for a total of 69–148 randomly selected regions from the acquired images (see the section on Methods) were presented as the mean ± SE and analyzed using one-way ANOVA followed by Tukey's post-hoc test. *p <* *0.05, **p <* *0.01, ***p <* *0.001, and ****p <* *0.0001.

As neutrophils come in direct contact with HUVECs inside the blood vessel but not the ECM, we first tested the elastic modulus of the HUVEC monolayer on varied substrate stiffness following a recent atomic force microscopy (AFM) protocol. Here, a quantitative nanomechanics (QNM) mode of AFM allowed us to examine the height, the topography and the elastic modulus of the live HUVEC monolayer simultaneously [Fig. 2(a)]. The obtained AFM-based elastic modulus map of the HUVEC monolayer revealed the clear mechanical differences of a single HUVEC cell on the cell body and the cell periphery. Under the same substrate stiffness, a higher elastic modulus was found for the cell periphery than for the cell body [Fig. 2(b)]. Moreover, both the regional [Fig. 2(b)] and global [Fig. 2(c)] elastic moduli increased with increasing substrate stiffness. These results implied that the variation of substrate stiffness is able to be sensed directly by the HUVECs placed on the top of the substrate, which in turn transmits these mechanical signals to the neutrophils that adhered to the HUVECs.

### Ca^2+^ spike in neutrophils is increased in a stiffness-dependent manner

**FIG. 3.**
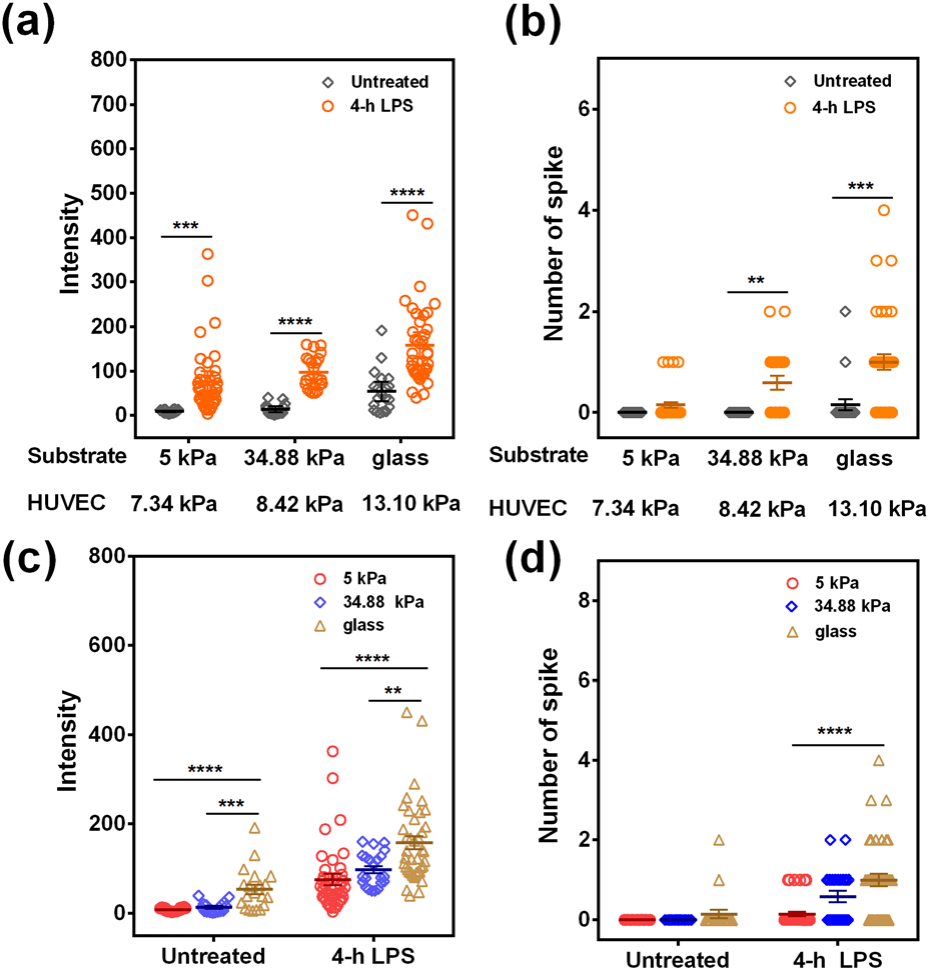
Stiffness-dependent Ca^2+^ spike in neutrophils on HUVECs. Ca^2+^ intensity (a) and spike number (b) within 25 min were compared between intact and LPS-treated cells, and data on Ca^2+^ intensity (c) and spike number (d) are presented among three stiffness values. Data were collected from at least 3 independent experiments and all the data from a total of 16–40 cells were presented as the mean ± SE. Data in (a) and (b) were compared using the *t*-test, and the replotted data in (c) and (d) were compared using one-way ANOVA followed by Tukey’s post-hoc test and presented as mean ± SE. **p <* *0.01, ***p <* *0.001, and ****p <* *0.0001.

Neutrophils were then added to settle down and adhere to the top of the 4-h LPS-treated HUVEC monolayer that was pre-formed on a thin PA gel [supplementary material, Fig. S2(a)], and the Ca^2+^ spikes in neutrophils were monitored by time-lapsed imaging [supplementary material, Figs. S2(b) and S2(c)]. By defining a specific Ca^2+^ spike event with Ca^2+^ intensity >50 and the spike frequency as the Ca^2+^ peak number within a 25-min duration [supplementary material, Figs. S2(d)–S2(f)], it was found that LPS treatment on HUVECs upregulated the Ca^2+^ intensity and the spike number in spreading neutrophils onto the HUVEC monolayer [Figs. 3(a) and 3(b)], implying that LPS-induced enhancement of adhesive molecules can mediate the Ca^2+^ spike. Furthermore, replotting these data on substrate stiffness suggested that the Ca^2+^ intensity and the spike number increased with increasing substrate stiffness [Figs. 3(c) and 3(d)], as seen in the statistical difference between glass (stiff substrate) and PA gel (soft substrate).

As shown in Fig. 1, the expression levels of P-selectin and ICAM-1 in the 5 kPa group are lower than those in the 35 kPa group. To elucidate if the molecule expressions act as the primary factor that differentially regulates the neutrophil Ca^2+^ on 5 kPa and 34.88 kPa gels [Figs. 3(c) and 3(d)], instead of the substrate stiffness, two strategies could be proposed. One is attempting to obtain similar molecule expressions on both 5 and 34.88 kPa gel by adjusting the LPS concentration used for stimulating the HUVECs on distinct stiffness. It is hard to achieve the goal experimentally, since varying LPS concentration also altered the elasticity of HUVECs (*data now shown*). An alternative way is to validate the fact that the expression of adhesive molecules on HUVECs outnumbers that of counterpart molecules on neutrophils, on which the molecular expressions on HUVECs could not be a primary factor. We have estimated the molecular densities of the ligands on HUVECs and of the receptors on neutrophils to test if the ligand expression on HUVECs is saturated (Fig. S3). Here, the spreading area of a single HUVEC cell was calculated *via* microscopic images on 5 kPa and 34.88 kPa and glass substrates by 4-h LPS treatment [Fig. S3(i)], and the total numbers of P-selectin and ICAM-1 molecules on a single HUVEC cell were determined *via* flow cytometry after being digested with trypsin [Figs. S3(a)–S3(d)], respectively. The molecular densities of P-selectins and ICAM-1s on HUVECs [*black bar* in Figs. S3(k) and S3(l)] on each substrate were then obtained by dividing the total number of those molecules of a single HUVEC cell [Figs. S3(b) and S3(d)] by the average spreading area of a single HUVEC cell [Fig. S3(i)]. Similarly, the numbers of PSGL-1s, CD44s and CD18s on a single neutrophil [Figs. S3(e)–S3(h)], the spreading area of a single neutrophil on different substrates *via* the HUVEC monolayer [Fig. S3(j)] and the molecular densities of CD18, PSGL-1, and CD44s on neutrophils [*white and dotted bars* in Figs. S3(k) and S3(l)] were also obtained separately. The results [Fig. S3(k)] indicated that, for ICAM-1s on HUVECs, although the expression level of ICAM-1s on the 5-kPa substrate is lower than that on the 34.88-kPa substrate, the corresponding β_2_ integrin-ICAM-1 interaction is sufficient enough to mediate calcium response in the same contact area, which is mainly governed by the receptors on neutrophils. For P-selectin [*black bar* in Fig. S3(l)], as its expression is very low (5.8, 4.5, and 4.0 molecule/*μ*m^2^ for 5 kPa, 34.88 kPa, and glass, respectively), its role in mediating calcium response in neutrophils on different substrates is approximately at the same level. Thus, the expression levels of P-selectins and ICAM-1s would not be a primary factor that differentially regulates the neutrophil Ca^2+^ on 5-kPa and 34.88-kPa gels, and such stiffness-dependent manner of calcium response is most likely mechanically specific.

Additionally, to exclude the potential impacts of the PA gel itself or the thickness of the gel on the measured fluorescence intensity of the calcium spike, fluorescein isothiocyanate (FITC)-labeled polystyrene beads were placed on the three substrates separately and their fluorescence intensities were compared. The results indicated that neither the gel itself nor the thickness of the gel would affect the fluorescent signals [Fig. S4], convincing the data collected above.

### Stiffness-dependent β_2_-integrin activation by selectin-ligand engagement is critical for varied Ca^2+^ spike in neutrophils

**FIG. 4.**
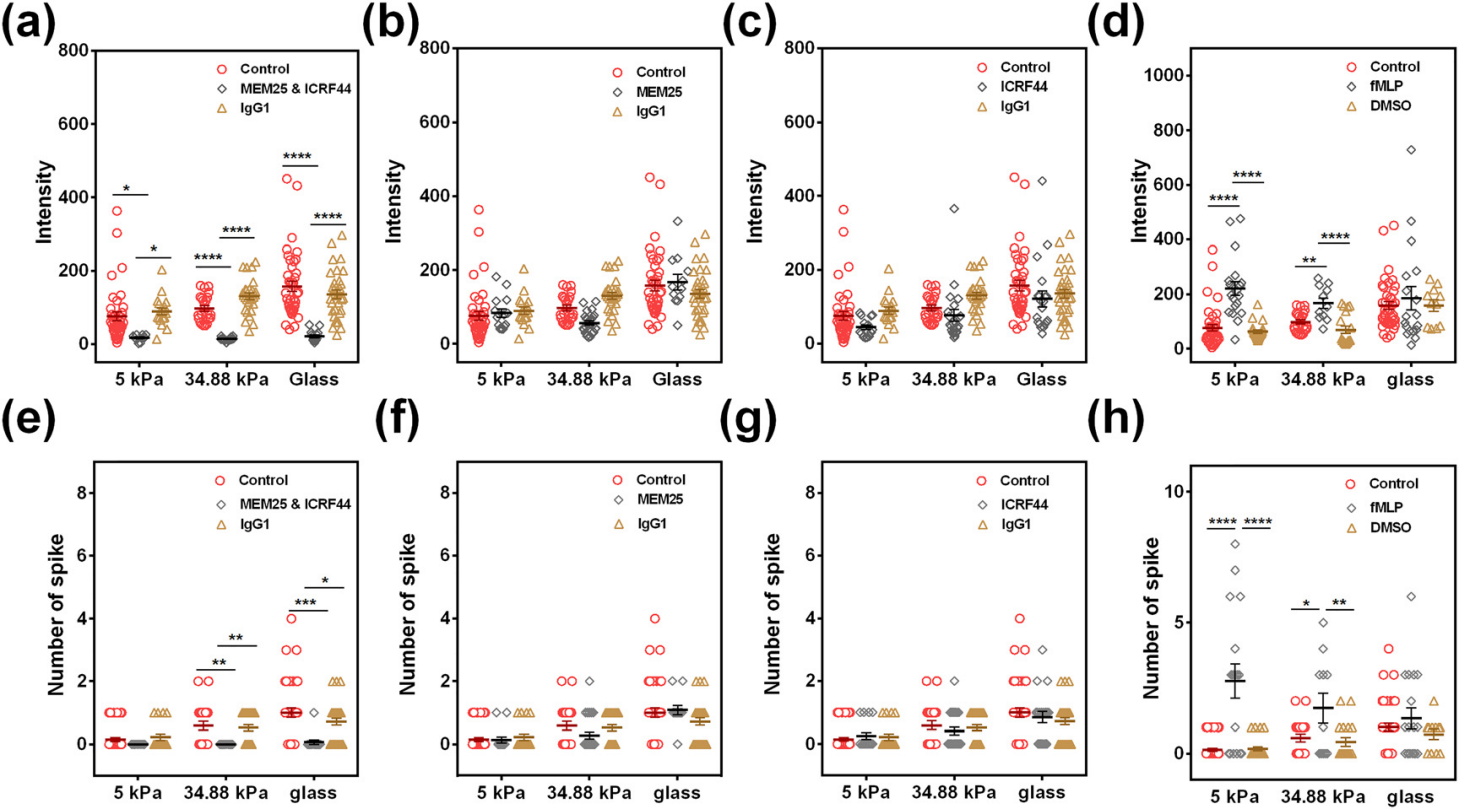
Active β_2_-integrins are critical for stiffness-dependent Ca^2+^ spike in neutrophils on 4-h LPS-treated HUVECs. Ca^2+^ intensity (a)–(d) and the spike number (e)–(h) in neutrophils were compared at three different stiffnesses when β_2_-integrins were blocked by 5 *μ*g/ml of anti-CD11a (MEM25) and anti-CD11b (ICRF44) mAbs [(a) and (e)], 5 *μ*g/ml of anti-CD11a (MEM25) alone [(b) and (f)]. 5 *μ*g/ml of anti-CD11b (ICRF44) mAbs alone [(c) and (g)], or activated by 10 *μ*M fMLP [(d) and (h)]. Isotype-matched irrelevant mAbs (IgG1, 5 *μ*g/ml) [(a)–(c) and (e)–(g)] and DMSO [(d), (h)] serve as control, respectively. Data were collected from at least 3 independent experiments. All the data for a total of 11–40 cells are presented as mean ± SE under different conditions and analyzed using one-way ANOVA followed by Tukey's post-hoc test. *p <* *0.05, **p <* *0.01, ***p <* *0.001, and ****p <* *0.0001.

Activation of β_2_-integrins is known to mediate neutrophil rolling and adhesion on endothelium.^19,20^ It is also noticed that the presence of E-/P-selectins on HUVECs can activate β_2_-integrins in neutrophils,^21,22^ and the tensile forces exerted on high-affinity LFA-1 specifically induce intracellular calcium response.^23,24^ To test whether active β_2_-integrins are responsible for stiffness-dependent Ca^2+^ spike, we tested Ca^2+^ flux in neutrophils by blocking or activating β_2_-integrins. Cooperative blockage of β_2_-integrins using anti-CD11a (MEM25) and anti-CD11b (ICRF44) mAbs dramatically reduced Ca^2+^ intensity to the basal level and completely abolished the spike at all stiffnesses [Figs. 4(a) and 4(e)], suggesting that β_2_-integrins dominate Ca^2+^ spike in neutrophils. Intriguingly, individual blockage of β_2_-integrins using MEM25 [Figs. 4(b) and 4(f)] or ICRF44 mAbs [Figs. 4(c) and 4(g)] alone almost had no effects on either the peak intensity [Figs. 4(b) and 4(c)] or the peak number [Figs. 4(f) and 4(g)]. By contrast, activating CD 18 *via* formylmethionyl leucyl phenylalanine (FMLP) stimulation significantly upregulated the intensity and the peak number on PA gels at 5 and 34.88 kPa [Figs. 4(d) and 4(h)], but had no effect on glass. This observation indicated that the stiffest glass substrate could promote β_2_-integrin activation to the similar level as fMLP did, and the β_2_-integrin activation induced Ca^2+^ spike *via* fMLP is no longer effective on glass. Moreover, antibody-induced inhibition or fMLP-induced activation of β_2_-integrin is specific since the incubation with isotype-matched irrelevant mAbs IgG1 [Figs. 4(a)–4(c) and 4(e)–4(g)] and vehicle control dimethyl sulfoxide (DMSO) [Figs. 4(d) and 4(h)] had no effects on Ca^2+^ spike. These results indicated that active β_2_-integrin is responsible for stiffness-dependent Ca^2+^ spike in neutrophils on the 4-h LPS-treated HUVEC monolayer.

**FIG. 5.**
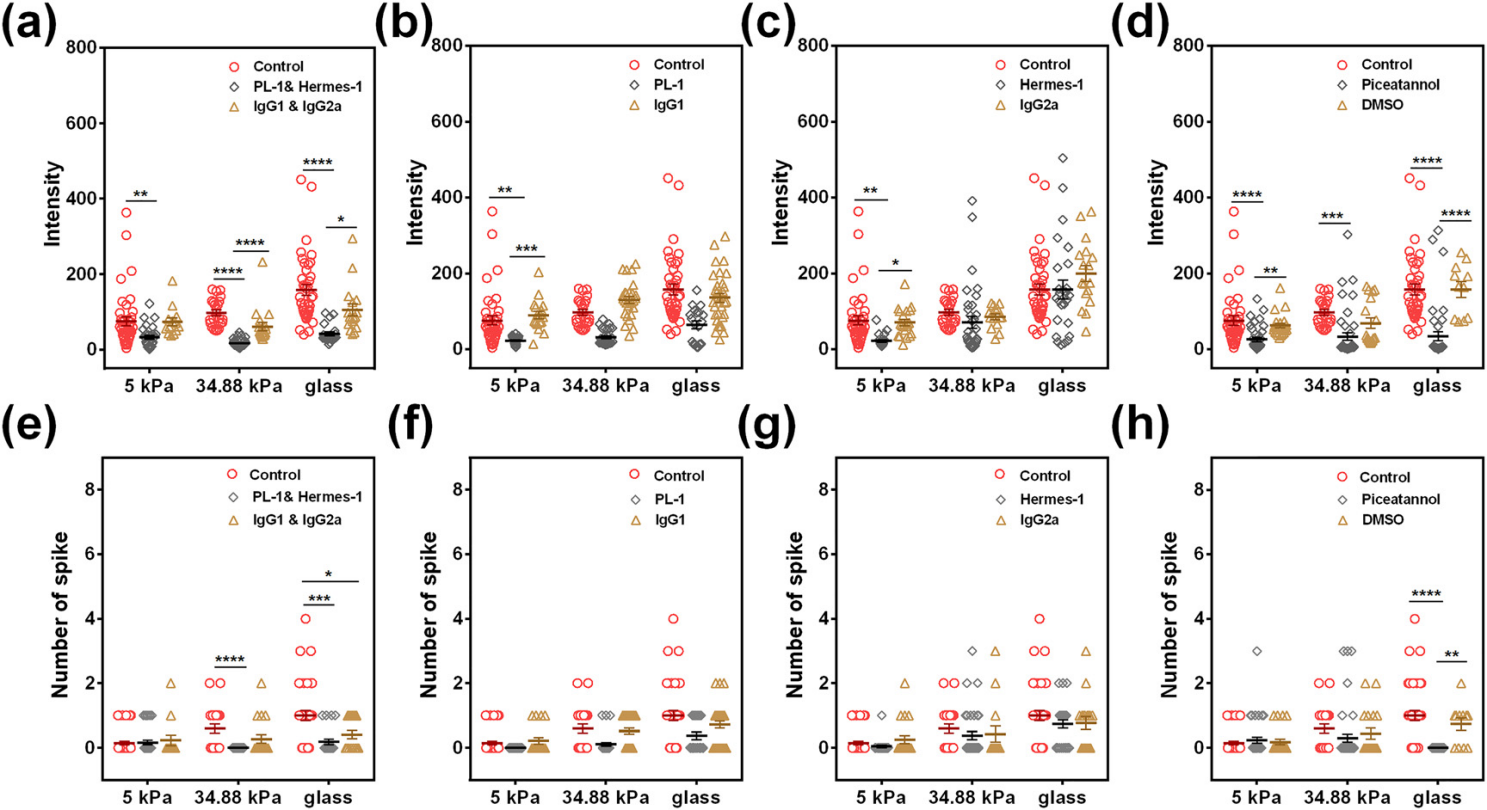
Selectin-induced β_2_-integrin activation *via* the Syk signaling pathway is responsible for stiffness-dependent Ca^2+^ spike in neutrophils on 4-h LPS-treated HUVECs. Ca^2+^ intensity (a)–(d) and spike number (e)–(h) of neutrophils were compared for three different stiffnesses when both CD44 and PSGL-1 were blocked by their respective blocking mAbs (5 *μ*g/ml) [(a) and (e)], 5 *μ*g/ml of PL-1 alone [(b) and (f)], and 5 *μ*g/ml of Hermes-1 alone [(c) and (g)]. Isotype-matched irrelevant mAbs IgG1 and IgG2a (5 *μ*g/ml) serve as control. 20 *μ*M Piceatannol [(d), (h)] was used to inhibit the signaling pathway of selectin-induced β_2_-integrin activation. DMSO serves as vehicle control. Data were collected from at least 3 independent experiments. All the data for a total of 13–40 cells are presented as mean ± SE under different conditions and analyzed using one-way ANOVA followed by Tukey's post-hoc test. *p <* *0.05, **p <* *0.01, ***p <* *0.001, and ****p <* *0.0001.

As ligand-engaged P- or E-selectins can activate β_2_-integrins,^25,26^ we hypothesized that P- and E-selectin-induced β_2_-integrin activation mediates Ca^2+^ spike in neutrophils on 4-h LPS-treated HUVECs. Thus, we further tested the related molecular mechanisms for two physiological selectin ligands on the neutrophil surface, PSGL-1 and CD44, where PSGL-1 binds to P-selectin and E-selectin^27^ and CD44 serves as an E-selectin ligand.^28,29^ Blocking both the CD44s and PSGL-1s [Figs. 5(a) and 5(e)] reduced the Ca^2+^ intensity to the basal level in all stiffnesses and lowered the peak except for the case at 5 kPa, diminishing their stiffness dependence. Similarly, individual blockage of PSGL-1 using PL-1 [Figs. 5(b) and 5(f)] or blockage of CD44 using Hermes-1 [Figs. 5(c) and 5(g)] alone had almost no effects on either the peak intensity [Figs. 5(b) and 5(c)] or the peak number [Figs. 5(f) and 5(g)]. Isotype-matched irrelevant mAbs IgG2a and IgG1 [Figs. 5(a) and 5(e)] had no effects. These data indicated that ligand-engaged selectins are involved in stiffness-dependent Ca^2+^ spike in neutrophils mediated by active β_2_-integrins.

It is known that the above β_2_-integrin activation *via* selectin ligand engagement is present in a Syk-dependent manner,^25,26^ and we further used piceatannol, but not DMSO, to inhibit selectin-induced β_2_-integrin activation through the Syk/Btk signaling pathway. Such an inhibition significantly decreased Ca^2+^ intensity to the basal level at all stiffnesses [Fig. 5(d)], while the spike number was not altered on PA gels at 5 or 34.88 kPa but was completely abolished on glass [Fig. 5(h)], thus diminishing their stiffness dependence. As E-selectins can activate β_2_-integrin binding to ICAM-1 through the Src/mitogen activated protein kinase (MAPK) signal transduction pathway,^18^ we also tested the role of Src signaling on selectin-induced β_2_-integrin activation by inhibiting Src *via* PP1. The data so-collected [Fig. S5] were the same as those by inhibiting Syk. Taken together, these results suggested that selectin-induced β_2_-integrin activation is responsible for stiffness-dependent Ca^2+^ spike in neutrophils on a 4-h LPS-treated HUVEC monolayer.

### Cytoskeletal remodeling is responsible for stiffness-dependent Ca^2+^ spike in neutrophils

**FIG. 6.**
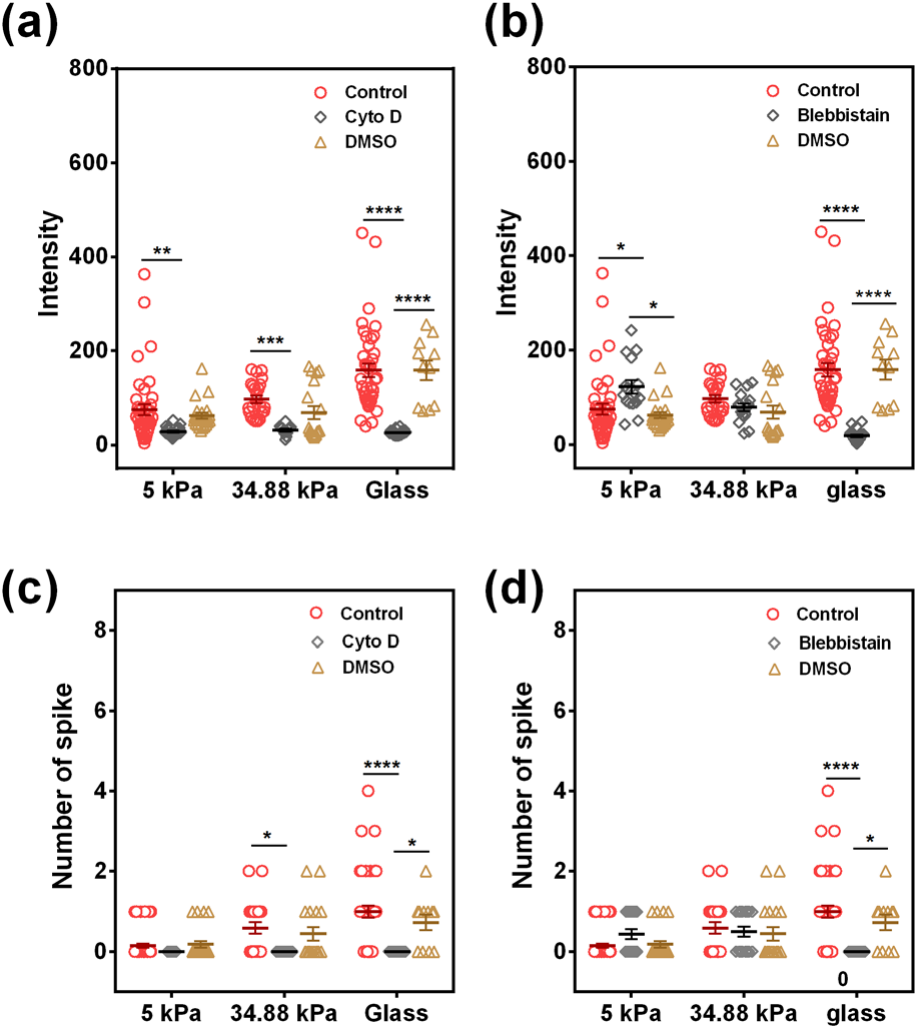
Cytoskeletons are involved in regulating Ca^2+^ spike in neutrophils on 4-h LPS-treated HUVECs. Ca^2+^ intensity [(a) and (b)] and spike number [(c) and (d)] of neutrophils were compared at three different stiffnesses when Cyto D (5 *μ*M, 15 min) [(a) and (c)] or blebbistatin (15 *μ*M, 15 min) [(b) and (d)] was used to pretreat the neutrophils for inhibiting the involvement of actin filament and myosin II, respectively. DMSO serves as vehicle control. Data were collected from at least 3 independent experiments. All the data for a total of 11–40 cells are presented as mean ± SE under different conditions and analyzed using one-way ANOVA followed by Tukey's post-hoc test. *p <* *0.05, ***p <* *0.001, and ****p <* *0.0001.

As substrate stiffness is known to affect the cell morphology, the cytoskeletal structure and the cell adhesion,^30^ and adherent cells can generate contractile forces by actin polymerization and actomyosin contractile machinery,^31^ it is reasonably hypothesized that the cytoskeleton and the intracellular mechanical tension may mediate Ca^2+^ signals in sensing the substrate stiffness. To investigate the roles of the cytoskeletal network, cytochalasin D (Cyto D) was applied to depolymerize actin filaments. As shown in Figs. 6(a) and 6(c), disrupting actin filaments had great effects on Ca^2+^ spike in neutrophils in all stiffnesses and both Ca^2+^ intensity and peak number were significantly inhibited by Cyto D treatment. These results suggested that intracellular cytoskeletal support through actin filaments is necessary for the induction of stiffness-dependent Ca^2+^ spikes. Surprisingly, inhibiting myosin II using blebbistatin [Figs. 6(b) and 6(d)] only attenuated the Ca^2+^ intensity and the peak number on the glass substrate and had no effects on Ca^2+^ spike on PA gels at 5 kPa and 34.88 kPa substrates. Collectively, these results implied that cytoskeletal support is essential for Ca^2+^ spike, and active actomyosin contractility may be more sensitive to substrate stiffness that is responsible for tension-induced Ca^2+^ spike in neutrophils on glass.

### Tension-activated Ca^2+^ channels play key roles in initiating stiffness-dependent Ca^2+^ response

**FIG. 7.**
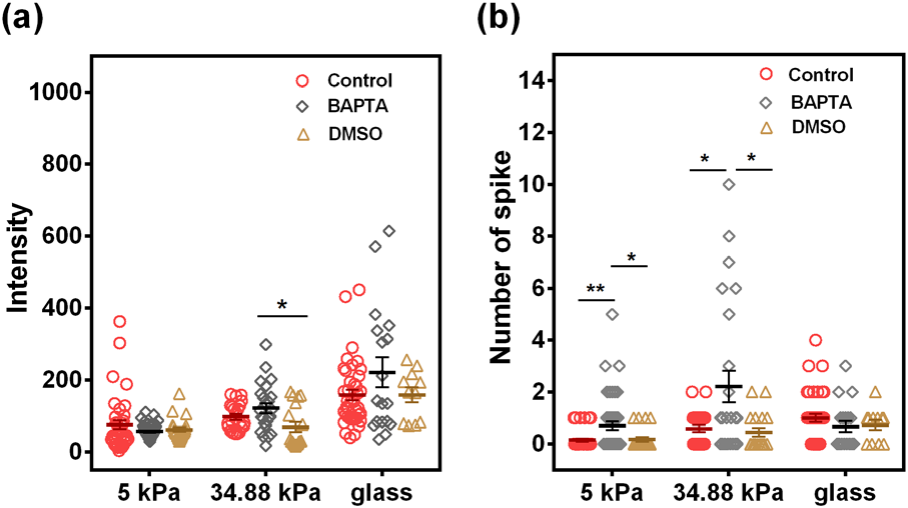
Chelating intracellular Ca^2+^ is not effective in stiffness-dependent Ca^2+^ spike in neutrophils. Intracellular Ca^2+^ in neutrophils is chelated using BAPTA (20 *μ*M), and Ca^2+^ intensity (a) and spike number (b) are presented. DMSO serves as a vehicle control. Data were collected from at least 3 independent experiments. All the data for a total of 11–43 cells are presented as mean ± SE under different conditions and analyzed using one-way ANOVA followed by Tukey's post-hoc test. *p <* *0.05 and **p <* *0.01.

Cytoplasmic Ca^2+^ spike is usually governed by Ca^2+^ trafficking between the plasma membrane and the endoplasmic reticulum (ER) membrane. Using 1,2-bis(o-aminophenoxy)ethane-N,N,N′,N′-tetraacetic acid (BAPTA) to chelate intracellular Ca^2+^ seemingly had no effects on Ca^2+^ intensity and spike number of stiffness-dependent Ca^2+^ spike, especially for neutrophils on glass [Figs. 7(a) and 7(b)]. This observation indicated that Ca^2+^ influx from the extracellular environment dominates the stiffness-dependent Ca^2+^ spike in neutrophils.

**FIG. 8.**
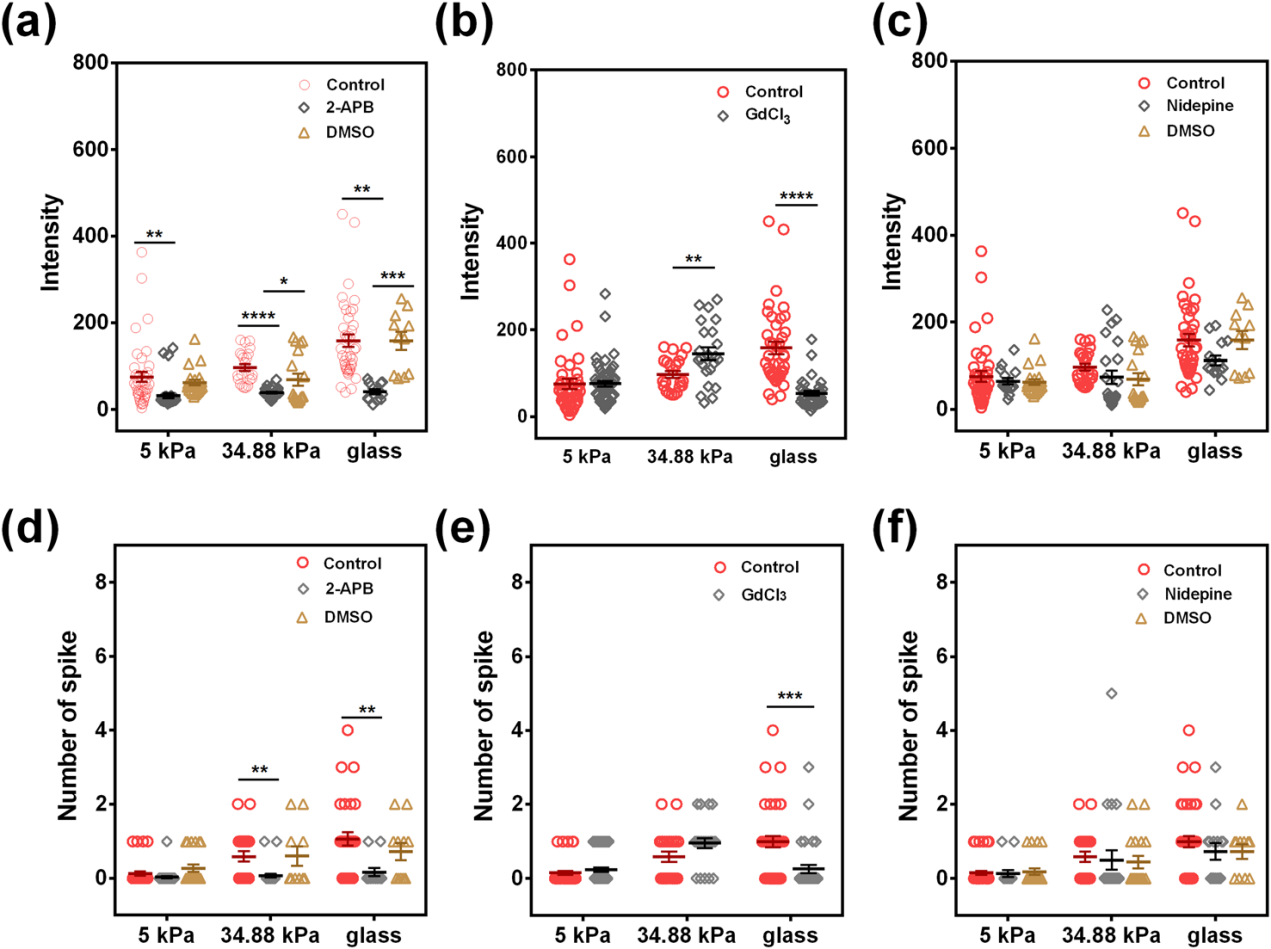
Stiffness-dependent Ca^2+^ spike depends on calcium entry *via* the plasma membrane. Ca^2+^ intensity (a)–(c) and peak number (d)–(f) in neutrophils were compared at different stiffnesses when 50 *μ*M 2-APB (a) and (d), 5 *μ*M GdCl_3_ (b) and (e), and 10 *μ*M Nifedipine (c) and (f) were used to inhibit calcium entry *via* plasma membrane channels. DMSO serves as a vehicle control. Data were collected from at least 3 independent experiments. All the data for a total of 11–56 cells are presented as mean ± SE under different conditions. Data in (a), (c), (d) and (f) were analyzed using one-way ANOVA followed by Tukey's post-hoc test. Data in (b) and (e) were analyzed using t-test. *p <* *0.05, **p <* *0.01, ***p <* *0.001, and ****p <* *0.0001.

To examine whether calcium influx from the extracellular environment is involved in the stiffness-dependent Ca^2+^ spike, we first used 2-amino-ethoxydiphenylborate (2-APB) to inhibit IP3R proteins on the membrane and found that inhibiting IP3R lowered Ca^2+^ intensity and peak at all stiffnesses [Figs. 8(a) and 8(d)], indicating that the store-operated Ca^2+^ (SOC) channel plays an important role in Ca^2+^ spike. We further applied a potent inhibitor of tension-activated calcium permeable channels, gadolinium chloride (GdCl_3_), or an inhibitor of L-type Ca^2+^ channel, Nifedipine. As shown in Figs. 8(b), 8(c), 8(e), and 8(f), GdCl_3_ specifically inhibited Ca^2+^ intensity and spike number in neutrophils on glass, whereas inhibiting the L-type Ca^2+^ channel almost had no effect on Ca^2+^ spike in neutrophils. Taken together, our results suggested that Ca^2+^ entry *via* tension-activated calcium permeable channels from the extracellular environment upon substrate stiffness is essential for the stiffness-dependent Ca^2+^ spike in neutrophils.

## DISCUSSION

**FIG. 9.**
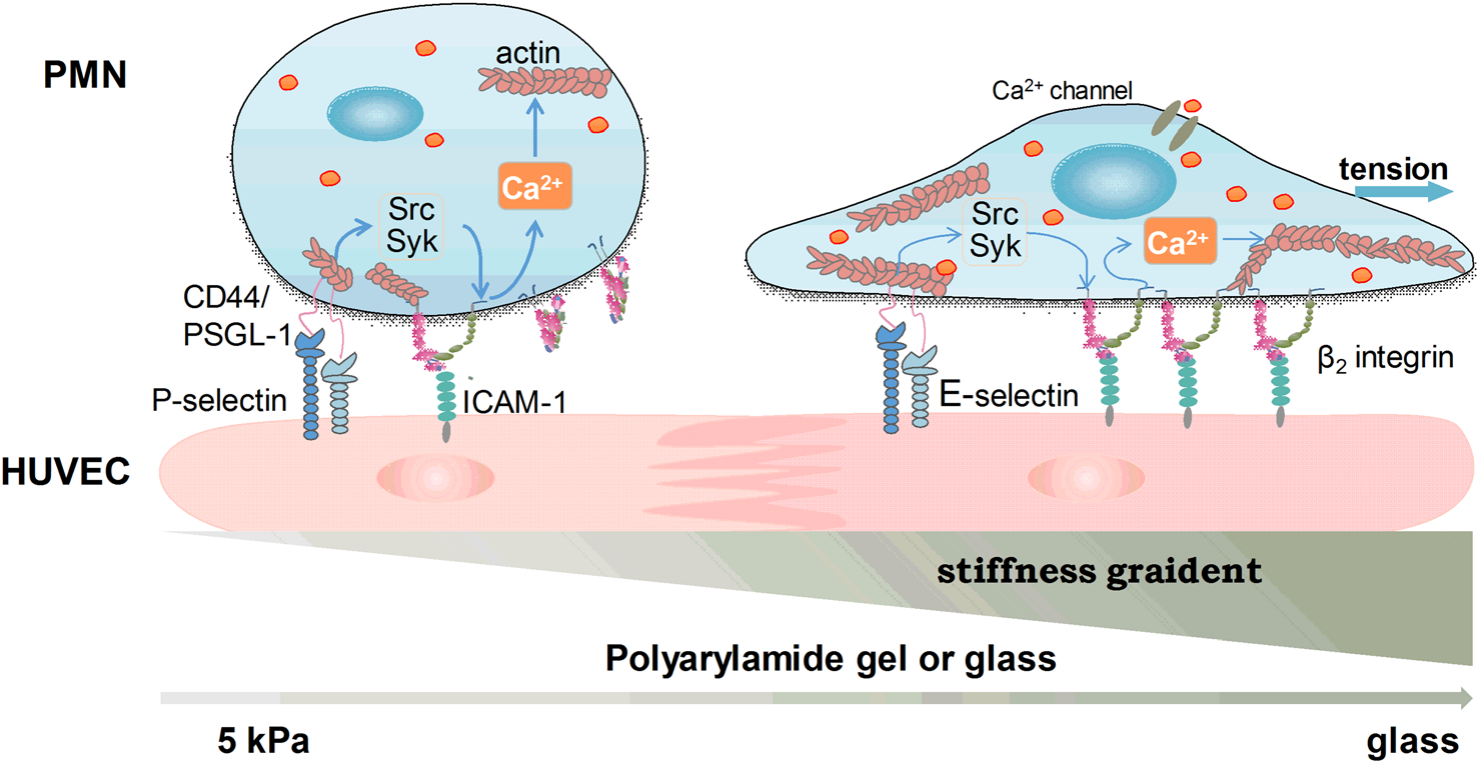
Working model of stiffness-dependent calcium response for neutrophils on HUVECs. Selectin-induced β_2_-integrin activation through the Syk/Src signal pathway is dominant in initiating neutrophil calcium responses. A rise in cytoplasmic Ca^2+^ concentration may activate calcium-sensitive secondary Rho GTPases to direct actin polymerization and actomyosin contraction. The high affinity β_2_-integrin bonds provide a physical linkage between the ECM and the actin network. Substrate stiffness applies tension forces through β_2_-integrin bonds and directly opens the stretch-activated calcium channels on the plasma membrane.

In this work, we built up an *in vitro* cellular inflammation model to elucidate the impact of substrate stiffness on Ca^2+^ spike in neutrophils adhering to HUVECs that are pre-layered on varied substrates. A complicated, coordinated feature of Ca^2+^ response regulation in neutrophils was observed, in which selectin-induced β_2_-integrin activation *via* the Syk/Src signaling pathway is dominant in initiating the neutrophil Ca^2+^ flux, and a rise in cytoplasmic Ca^2+^ concentration may activate Ca^2+^-sensitive secondary kinases (such as Rho GTPases) to direct actin polymerization and actomyosin contraction.^23^ The high affinity β_2_-integrin-ICAM-1 bonds provide a physical linkage between the ECM and the actin cytoskeleton, and thus play a critical role in cellular mechano-transduction.^31,32^ Substrate stiffness applies tension forces *via* β_2_-integrin bonds to directly activate those tension-sensitive Ca^2+^ channels on the plasma membrane. A working model was proposed to illustrate how Ca^2+^ spike in neutrophils is regulated by substrate stiffness *via* adhering to the HUVEC monolayer (Fig. 9).

Substrate stiffness has a significant impact on cellular behaviors for both endothelial cells and neutrophils. The stiffness of the basement membrane (serving as an endothelial substrate) and of endothelium (as a leukocyte substrate) collectively contribute to the process of leukocyte transmigration. HUVEC morphology and neutrophil migration are regulated by substrate elasticity.^10–15^ However, most tests in the past decades are performed directly placing leukocytes on PA gels, ignoring the interplay between biological (from ECs) and mechanical (from substrate stiffness) microenvironments. In our work, the HUVEC monolayer was first placed on a PA gel substrate with different stiffnesses, acting as a leukocyte substrate. Using AFM technique, the deflection images (Fig. 2) were obtained and the Young’s modulus was estimated for control and 4-h LPS-treated HUVEC monolayers on a 5-kPa or 34.88-kPa PA gel or glass substrate, respectively (Fig. 2). The average modulus of intact HUVECs on all substrates is no more than 20 kPa (Figs. 2(b), 2(c), 3(a), and 3(b)]. The average modulus of 4-h LPS-treated HUVECs reaches a slightly increased peak at 13.10 kPa on glass but still stays below 10 kPa on two PA gels. These measurements indicated that, although the stiffness varies greatly from PA gel (∼kPa) to the glass substrate (∼GPa), its effect on endothelial cell elasticity is not as great as expected. This may partially explain why the stiffness dependence of calcium response is not so significantly different for the data between 5 vs. 34.88 kPa on PA gel compared to those on glass (Fig. 3). It is also noticed that the AFM tip type and radius affect the cell elasticity measured.^33^ To further confirm the so-obtained data on HUVEC moduli, we also measured the HUVEC monolayer modulus using a spherical tip. The results indicated that the HUVEC modulus yields 1.35, 1.45, and 1.77 kPa on 5- and 34.88-kPa PA gel and glass, which are comparable with the values in the literature.^13^ In addition, we also obtained the higher moduli in the HUVEC periphery than in the HUVEC body [Fig. 2(b)]. This is not inconsistent with the observation that the nucleus is the stiffest component of cells.^34^ In fact, what we measured here is the elasticity of the cytoplasmic region right above the nucleus, but not that of the isolated nucleus itself. The indentation depth is about 400 nm and the impact of the stiffer nucleus is not so evident, resulting in relatively lower values of the HUVEC body compared to the HUVEC periphery that is full of actin stress fibers. This finding is also in accordance with the observations that the cell periphery yields higher elasticity.^33,35–37^ Taken together, the *in vitro* model presented here is different from those by placing neutrophils directly onto PA gel.^15^ Our model is more physiologically relevant by linking ECM stiffness variation to neutrophil calcium response through the HUVEC monolayer (Figs. 1–3), especially for understanding the underlying mechanisms in cardiovascular diseases such as atherosclerosis.

Ca^2+^ spike plays an important role in functional responses of neutrophils. The cytosol Ca^2+^ level is balanced by the extracellular calcium influx and the ER-derived calcium release, which is associated with the Orail-3/STIM1–2 signaling pathway to promote neutrophil or lymphocyte recruitment in inflammation.^38–40^ Meanwhile, neutrophil calcium dynamics undergoes enriched leading-edge calcium flux for migrating neutrophils in *in vivo* wound healing or drug-induced chemotaxis.^16^ And, such calcium dynamics is correlated with neutrophil state transition among crawling, spreading, or agile morphologies.^41^ Functionally, integrin-associated intracellular Ca^2+^ spike triggers the disassembly of focal adhesion with similar kinetics of stress fiber end movement and disrupts integrin-ECM interactions, supporting a key role for Ca^2+^-sensitive inside-out signaling in cell migration.^42^ Thus, the calcium response in neutrophils displays a biologically and biomechanically coordinated feature. In the current work, similar coordination is demonstrated: the stiffness-dependent Ca^2+^ spike in neutrophils is mediated by selectin-induced high-affinity β_2_-integrins *via* Syk/Src signaling and actin/myosin II remodeling, which is governed by tension-related gating of multiple calcium channels from extracellular Ca^2+^ influx (Figs. 4–8 and S5). It should be pointed out that the underlying mechanisms of such stiffness-dependent calcium response in neutrophils could be sophisticated, as seen in the exceptional observation that chelating intracellular Ca^2+^ by BAPTA intensifies the Ca^2+^ influx on 34.88 kPa gel compared to those on either 5 kPa gel or glass (Fig. 7). While no stiffness dependences of BAPTA inhibition are found for various cell types and different substrates in the literature, the inhibition test in this study is just to confirm that intracellular Ca^2+^ release would not affect the calcium responses, and the calcium influx is transmitted through mechanically sensitive calcium channels into the cell. While the intracellular signaling pathways of calcium responses inside neutrophils are quite complicated, we briefly tested in the current work the potential roles of typical calcium ion channels using inhibition measurements (Figs. 7 and 8). Noticing that these inhibition tests of typical calcium ion channels can only serve as starting points for their mechanistic studies, future works need to be done, at least, in the following aspects: (1) More specific inhibition tests are required using specific antibodies, target gene silence or gene editing assays since the effects induced by the agents used in the current work are broad. (2) More functional readouts such as cell crawling and transmigration dynamics are needed to be performed since the calcium is just a secondary messenger for many cellular processes, but not specific for stiffness-induced neutrophil recruitment. (3) Coupling mechanisms of molecular expression and substrate stiffness at low molecular expressions on HUVECs need to be elucidated on the calcium signaling in neutrophils.

Cellular sensing to substrate rigidity is usually accomplished through integrins interacting with ECM components. It is known that substrate stiffness directs the mechanical activation of integrins binding to ECM through myosin-II-generated contractive force, leading to internal signaling *via* phosphorylation of focal adhesion (FA) kinase. And, cytoskeletal dynamics and MLCK-driven actomyosin contractility have a significant impact on cellular tension.^43^ We hypothesized that cytoskeletal support and active actomyosin contraction are all required for stiffness-dependent Ca^2+^ spike in neutrophils. Surprisingly, although depolymerizing the actin fibers *via* Cyto D completely abolished Ca^2+^ spike in all stiffness, inhibiting the myosin-II proteins *via* blebbistatin only decreased Ca^2+^ spike on glass (Fig. 6). These results suggested that the intracellular mechanical support derived from F-actin is necessary for Ca^2+^ spike in neutrophils in all stiffness, but the active actomyosin contraction is only required for tension-induced Ca^2+^ spike in neutrophils on stiff substrates, where neutrophils suffer high tension from actomyosin contraction. This is consistent with the observation of calcium permeable channels on the plasma membrane because inhibiting tension-activated calcium permeable channels *via* GdCl_3_ decreases only the intensity and the peak number in neutrophils on glass (Fig. 8), but not on relatively soft PA gel substrates at 5 or 34.88 kPa. These data are seemingly reasonable, since higher tension for those neutrophils placed on stiffer HUVECs, derived from the stiffest glass, provides high probability to open these Ca^2+^ channels. Interestingly, the spike number of Ca^2+^ spike in neutrophils seems more sensitive to substrate stiffness, as compared to Ca^2+^ intensity. This is in accordance with the expectation that the mechanically-induced calcium signals are encoded by the spike number, but not the amplitude, of Ca^2+^ spike.^44^ Evidently, the mechanisms of substrate stiffness encoding Ca^2+^ spike number needs to be further explored in the future studies. An exceptional result lies in the fact that blebbistatin enhances neutrophil Ca^2+^ signaling on 5 kPa, has no effect on 35 kPa, and eliminates signaling on glass [Figs. 6(b) and 6(d)]. While the exact mechanisms are not clear for this observation, future works are required to test the following possibility: on stiff glass, actomyosin contraction is high and blebbistatin can completely inhibit the actomyosin contraction-mediated integrin activation and in turn the calcium responses in neutrophils; on soft PA gel, actomyosin contraction is low and the enhancement of calcium signals in neutrophils after blebbistatin inhibition is presumably attributed to the fluctuation of weak signals.

It should also be pointed out that there are multiple cytokines or chemokines presented in leukocyte recruitment, which could possibly regulate the calcium response in neutrophils placed on HUVECs. For example, IL-1 or tumor necrosis factor-α (TNF-α) can enhance the expressions of E- or P-selectin expression on HUVECs, which could also manipulate the intensities of calcium responses.^45–48^ Thus, future works are required using other agents for understanding the generality of our findings from the LPS-induced effects.

Collectively, we presented a stiffness-dependent Ca^2+^ spike manner in neutrophils, which is mediated by mechanical regulation of substrate stiffness *via* layered HUVECs. Binding of E-/P-selectin-CD44/PSGL-1 activates β_2_-integrins to a high-affinity state and β_2_-integrin-ICAM-1 binding dominates the calcium responses in neutrophils *via* Syk/Src signaling. Multiple calcium ion channels are dispensable in this process, mainly attributed to the extracellular Ca^2+^ influx. This work provides an insight into elucidating Ca^2+^ spike in atherosclerosis from the viewpoint of mechanotransduction.

## METHODS

### Antibodies and reagents

Allophycocyanin (APC)-labeled anti-human CD44 (BJ18), Alexa Fluor® 647-labeled anti-human CD54 (HCD54), and phycoerythrin (PE)-labeled anti-human CD162 (KPL-1) monoclonal antibodies (mAbs), as well as isotype-matched irrelevant antibodies (Abs) LEAF™ purified mouse IgG1 (MOPC-21), purified rat IgG2a (RTK2758), and LEAF purified mouse IgM (MM-30) were obtained from BioLegend (San Diego, CA, USA). Mouse anti-human E-selectin (S9555) and anti-CD11a blocking mAbs (MEM25), as well as thymidine, heparin sodium, LPS, fMLP, cytochalasin D, PP1, piceatannol, blebbistatin, 2-amino-ethoxydiphenylborate (2-APB), gadolinium chloride (GdCl_3_), nifedipine and 1,2-bis(o-aminophenoxy)ethane-N,N,N′,N′-tetraacetic acid (BAPTA) were from Sigma-Aldrich (St. Louis, MO, USA). Anti-CD11b (ICRF44) and anti-PSGL-1 (PL-1) blocking mAbs were from Santa Cruz Biotechnology (Santa Cruz, CA, USA). Alexa Fluor 594-conjugated goat anti-mouse secondary Abs for immunofluorescence staining were from Abcam (Cambridge, MA, USA). FITC-labeled anti-P-selectin blocking mAbs (AK-6) were from Thermo Fisher Scientific (Rockford, IL, USA). Medium 199, Dulbecco’s Phosphate buffered Saline (DPBS), trypsin, and 4-(2-Hydroxyethyl)-1-piperazineethanesulfonic acid (HEPES) were from GE Healthcare Life Sciences (HyClone Laboratories, Logan, UT, USA). Amphotericin B was from Amresco (Solon, OH, USA), the basic fibroblast growth factor (bFGF) was from R&D Systems (Minneapolis, MN, USA) and fatal bovine serum (FBS) was from Gibco (Grand Island, NY, USA). Fluo-4-AM dye was from Invitrogen (Carlsbad, California, USA).

### Cells

The HUVEC line was purchased from American Type Culture Collection (ATCC, Manassas, VA, USA) and cultured in Medium 199 supplemented with 20% FBS, 100 U/ml penicillin, 20 mM HEPES, 3 *μ*g/ml thymidine, 1 mM L-glutamine, 14 U/ml heparin sodium, 25 *μ*g/ml amphotericin B, and 5 ng/ml bFGF. For experimental use, sub-cultured HUVECs up to passage 4 were plated on a PA gel substrate or glass coverslip to form a monolayer. Cells were then treated with LPS for 4 h to induce the expression of selectins and ICAM-1s.

Whole human blood was obtained from healthy human donors after the informed consent was obtained, as approved by the Animal and Medicine Ethics Committee of the Institute of Mechanics with the ethics approval ID number 2018–03, Chinese Academy of Sciences, in accordance with the Declaration of Helsinki. All the donors were unmedicated and selected randomly. Neutrophils were then isolated by a density gradient centrifugation protocol using Histopaque-1077 and Histopaque-1119 (Sigma-Aldrich), as previously described.^9^ Isolated neutrophils were suspended in ice-cold DPBS and kept in ice until use. The cells were then stained with Fluo-4-AM dye (Invitrogen, Carlsbad, California, USA) for monitoring their Ca^2+^ spike.

### Flow cytometry analysis and site density determination

Isolated neutrophils were incubated with 5 *μ*g/ml FITC-anti-human CD18, APC-anti-human CD44, PE-anti-human CD162 mAbs, PE-anti-human E-selectin, PE-anti-human P-selectin, Alexa Fluor 647-labeled anti-human CD54 or isotype-matched irrelevant mouse IgG1s and IgG2a, respectively, on ice for 45 min. Washed neutrophils were resuspended in DPBS and analyzed by a FACS Calibur cytometer (BD Biosciences, NJ, USA).

To determine the site densities of these adhesive molecules on HUVECs or neutrophils, the fluorescence intensities of the stained cells were quantified using standard fluorescence calibration beads (Quantum 25, Bangs Laboratories Inc., Fishers, IN, USA) to determine the mean number of molecules of equivalent soluble fluorochrome (MESF) per cell. The MESF value was divided by the F/P value of FITC-labeled mAbs and the surface area of the cell, and then converted into the site density of the target molecules.^49^

### Biopolymer substrate construction

Biopolymer substrates were constructed using PA hydrogel *via* a soft-contact lithography technique described in our previous work.^50,51^ Briefly, 10 ml of 40% acrylamide and 2% bis-acrylamide (Sigma-Aldrich, St. Louis, MO, USA) were mixed in water to form a solution with a constant concentration of 10% acrylamide and two varied concentrations of 0.03 and 0.30% bis-acrylamide. By adding 1/100 (v/v) of 10% ammonium peroxydisulfate (Amresco, Washington, USA) and 1/1000 (v/v) of *N,N,N*′*,N*′-tetramethylethylenediamine (Amresco, Washington, USA) into the mixture, 20 *μ*l of the mixture was quickly sucked with a pipette and transferred to a chloro-silanated, 35-mm glass-bottom dish (Nest Scientific, Rahway, NJ, USA). The glass bottom was covered with one 18-mm diameter circular glass to allow the gel to be polymerized for 10 min and rinsed with distilled H_2_O for at least 4 h. After adding 0.2 mg/ml sulfo-SANPAH solution (cross-linking agent) in an adequate volume, the mixture was irradiated with ultra-violet light for 25 min to form a planar PA hydrogel. Two sets of concentrations were used for 25% acrylamide + 15% bis-acrylamide (34.88 kPa) and 25% acrylamide + 1.5% bis-acrylamide (5 kPa), where the Young’s modulus of the gel was determined using a self-weighing assay.^50,51^ The gel with 100 *μ*m in thickness was coated with collagen I (2 ml of 20 *μ*g/ml) prior to seeding the cells. A glass coverslip with the same diameter and thickness was used as control.

### AFM test

Topographical and modulus images were scanned with a Dimension Icon atomic force microscope (Bruker, Bioscope Catalyst, Billerica, MA, USA) using the PeakForce Quantitative Nanomechanics (QNM) mode. The scanning size was 100 × 100 *μ*m^2^ with a resolution of 512 × 512 pixels, and a 0.15 Hz scanning rate was used. An AFM force curve of the target cell was obtained by indenting the cell surface using AFM probes equipped with a Nanoscope V controller and Nanoscope software version 8.15. The QNM-Live Cell (PFQNM-LC) probes (Bruker AFM Probes, Camarillo, CA, USA) with a spring constant of 0.07 N/m, a tip length of 17 *μ*m, and a tip radius of 65 nm were used. For all the measurements, an indentation depth of 400–500 nm was applied to determine the local cell elasticity. The probe’s spring constant was pre-calibrated before each measurement using the thermal fluctuation protocol with Bruker’s polystyrene test samples, as described previously.^52,53^ The cell modulus was estimated from fitting the collected force-displacement curves with the Sneddon model using manufacturer’s software (NanoScope Analysis, Bruker, MA, USA). With the QNM mode, the entire surface of a HUVEC cell was scanned point-by-point to obtain the elastic modulus value at high resolution, and the three-dimensional cell elasticity map was then generated for further analysis. In some cases, a spherical probe (NOVASCAN AFM probe, Boone, IA, USA) with a spring constant of 0.07 N/m and a tip radius of 2.5 *μ*m was also used to convince the robustness of HUVEC moduli measured, and the cell modulus was estimated from fitting the collected force-displacement curves with the Hertz model. All measurements were carried out in HUVEC culture medium at room temperature. On each PA gel or glass, at least six scanning fields (100 × 100 *μ*m^2^ with 512 × 512 pixels) were examined in three independent repeats. To reduce the labor consumption, a region of 25 *μ*m^2^ was randomly selected from each scanned image to calculate the average Young’s modulus in this region for further data analysis. All data were analyzed with Nanoscope Analysis version 1.8.

### Real time fluorescence imaging of intracellular calcium in neutrophils

100 *μ*l of HUVEC suspension with ∼5 × 10^4^ cells was placed onto PA gel or glass coverslip pre-coated with collagen I overnight to form a monolayer. After 4-h of LPS-treatment, the HUVEC monolayer was washed twice with PBS and replaced with culture media. Human neutrophils were loaded with 1.25 *μ*g/ml of Fluo-4-AM for 15 minutes at a concentration of 5 × 10^5^/ml in calcium-free PBS at 4 °C as described previously.^54^ Following loading, neutrophils were pelleted (centrifuged for 30 s at 500 g) and resuspended in PBS for use. Fluo-4-AM-stained neutrophils (5 × 10^5^) were added onto the HUVEC monolayer and allowed to adhere for 5 min before imaging. Cells were imaged on an automatic inverted microscope (IX81, Olympus, Japan) equipped with an electron-multiplying charge-coupled device (EMCCD) camera (897, Andor, UK), that was controlled by MetaMorph software (Universal Imaging, West Chester, Pa.). To image the calcium response in those adhered neutrophils on HUVECs, the Fluo-4-AM loaded neutrophils were excited by a 100-W xenon lamp through a 480AF30 filter (Omega Optical Inc., Brattleboro, Vt.) and a 60×/NA1.35 oil immersion objective lens was used to observe the changes in intracellular calcium in individual neutrophils on HUVECs at a 500-ms interval for 25 min. Here, the dish containing the cells was placed in a custom-made heating device that provides temperature control (37 ± 0.5 °C) and a 5% CO_2_ supply during live cell imaging.

In some cases, FITC-labeled 7.1-*μ*m diameter polystyrene beads (Bangs Laboratories, Inc., Fishers, IN, USA) were placed on the three substrates, excited by a 100-W xenon lamp *via* a 480AF30 filter (Omega Optical Inc., Brattleboro, Vt.), and imaged through a 60×/NA1.35 oil immersion objective lens (the same setup as intracellular calcium acquisition). Three independent repeats are performed and at least three FOVs are acquired in each repeat. Collected images are analyzed using the same protocol described above.

### Immunofluorescence staining

For immunostaining experiments, HUVECs cultured on a PA hydrogel or glass coverslip were rinsed in PBS and fixed in 4% paraformaldehyde at room temperature (RT) for 10 min. After blocking non-specific epitopes using 1% bovine serum albumin (BSA) (Sigma-Aldrich, St. Louis, MO) at 37 °C for 1 h, the collected cells were stained with primary mAbs at 37 °C for 1 h, incubated at 4 °C overnight, and rinsed five to seven times with PBS. Then, the adequate amount of labeled secondary Abs in 1% BSA/PBS was added and incubated at 37 °C for 1 h. After being washed five to seven times with PBS, the collected samples were incubated with Hoechst 33342 for 10–15 min at RT, washed three to five times with PBS, and then stored at 4 °C for examination by confocal laser scanning microscopy (Zeiss L710, Germany) with a 40×/0.95 NA objective. All images were analyzed using ImageJ (National Institute of Health, Bethesda, MD, USA).

### Image acquisition and data analysis

A custom-developed automated image analysis method was used to track the movement of a single neutrophil and quantify the Ca^2+^ signals of a single cell.^55^ In brief, for a single field of view (FOV), the average fluorescence intensity of one circular area without neutrophils was quantified as the background signal and subtracted from the calcium signals in Fluo-4-AM loaded neutrophils. Otsu’s method^56^ was used to calculate the threshold of intensity for segmenting and identifying the individual neutrophils from the surrounding background, since the fluorescent images recorded using Fluo-4-AM were bright and readily isolated from the background. The average intensity of a single cell was obtained by subtracting the acquired calcium signals by background signals and then averaging all the values over the entire region of the cell. While the typical real-time calcium dynamics in neutrophils exhibited a stable, low level of intensity spanning over the entire observation window with occasional spikes varying in amplitude, there are still multiple small spikes with very low intensities after background subtraction (*black arrows* in Fig. S2). To protect the potential overestimation of calcium signals, a threshold value of 50 was set in the current work to explicit those remarkable calcium spikes in neutrophils. For simplicity, a spike with the peak value over 50 was counted as a specific event (*red arrows* in Fig. S2). The number of spiking Ca^2+^ peaks was counted as the corresponding peak number in each neutrophil and then averaged for all the cells counted. Also, it was noticed that no photobleaching of calcium signals was found within the observation window in the current experimental settings.

### Statistical analysis

Data are presented as mean ± SE under different conditions. Statistical tests were performed using unpaired two-tailed Student’s *t* test between paired data or one-way ANOVA analysis followed by Tukey’s post-hoc test for grouped data. Prism statistical software (GraphPad Software, CA, USA) was used by setting statistical difference as *p <* *0.05, **p <* *0.01, ***p <* *0.001, and **** p <* *0.0001.

## SUPPLEMENTARY MATERIAL

See supplementary material for Fig. S1–S5.Fig. S1 indicates that selectin or ICAM-1 expression is increased on the 4-h LPS-treated HUVEC monolayer compared to that for untreated cells. Figure S2 illustrates the stiffness-dependent Ca^2+^ spike in neutrophils on 4-h LPS-treated HUVECs with the definition of Ca^2+^ spike in a neutrophil. Figure S3 compares the expressions of cellular adhesive molecules on HUVECs and neutrophils on three distinct substrates. Figure S4 presents the mean fluorescence intensity of FITC-labeled polystyrene beads on three substrates. Figure S5 demonstrates that the Src signaling pathway is responsible for stiffness-dependent Ca^2+^ spike in neutrophils on 4-h LPS-treated HUVECs.
